# Chaperonin Containing T-Complex Polypeptide Subunit Eta (CCT-eta) Is a Specific Regulator of Fibroblast Motility and Contractility

**DOI:** 10.1371/journal.pone.0010063

**Published:** 2010-04-30

**Authors:** Latha Satish, Sandra Johnson, James H-C. Wang, J. Christopher Post, Garth D. Ehrlich, Sandeep Kathju

**Affiliations:** 1 Center for Genomic Sciences, Allegheny-Singer Research Institute, Allegheny General Hospital, Pittsburgh, Pennsylvania, United States of America; 2 MechanoBiology Laboratory, Department of Orthopaedic Surgery, University of Pittsburgh School of Medicine, Pittsburgh, Pennsylvania, United States of America; Ohio State University, United States of America

## Abstract

Integumentary wounds in mammalian fetuses heal without scar; this scarless wound healing is intrinsic to fetal tissues and is notable for absence of the contraction seen in postnatal (adult) wounds. The precise molecular signals determining the scarless phenotype remain unclear. We have previously reported that the eta subunit of the chaperonin containing T-complex polypeptide (CCT-eta) is specifically reduced in healing fetal wounds in a rabbit model. In this study, we examine the role of CCT-eta in fibroblast motility and contractility, properties essential to wound healing and scar formation. We demonstrate that CCT-eta (but not CCT-beta) is underexpressed in fetal fibroblasts compared to adult fibroblasts. An *in vitro* wound healing assay demonstrated that adult fibroblasts showed increased cell migration in response to epidermal growth factor (EGF) and platelet derived growth factor (PDGF) stimulation, whereas fetal fibroblasts were unresponsive. Downregulation of CCT-eta in adult fibroblasts with short inhibitory RNA (siRNA) reduced cellular motility, both basal and growth factor-induced; in contrast, siRNA against CCT-beta had no such effect. Adult fibroblasts were more inherently contractile than fetal fibroblasts by cellular traction force microscopy; this contractility was increased by treatment with EGF and PDGF. CCT-eta siRNA inhibited the PDGF-induction of adult fibroblast contractility, whereas CCT-beta siRNA had no such effect. In each of these instances, the effect of downregulating CCT-eta was to modulate the behavior of adult fibroblasts so as to more closely approximate the characteristics of fetal fibroblasts. We next examined the effect of CCT-eta modulation on alpha-smooth muscle actin (α-SMA) expression, a gene product well known to play a critical role in adult wound healing. Fetal fibroblasts were found to constitutively express less α-SMA than adult cells. Reduction of CCT-eta with siRNA had minimal effect on cellular beta-actin but markedly decreased α-SMA; in contrast, reduction of CCT-beta had minimal effect on either actin isoform. Direct inhibition of α-SMA with siRNA reduced both basal and growth factor-induced fibroblast motility. These results indicate that CCT-eta is a specific regulator of fibroblast motility and contractility and may be a key determinant of the scarless wound healing phenotype by means of its specific regulation of α-SMA expression.

## Introduction

Adult mammalian tissues respond to injury by healing with scar formation [1], [2]; in contrast, mammalian fetuses demonstrate an ability to heal without scar, a process that has been likened to regeneration [3], [4]. Although scar formation allows for the rapid sealing of an injured area, it can frequently prove the source of persistent pathology in the organism, eg. restricting movement, narrowing viscera etc. At the phenotypic level adult and fetal wound healing differ in multiple important respects: adult wound healing is marked by a prominent initial acute inflammatory response, which is absent in fetal wound healing, and fetal wound healing displays no accumulation of intermediary granulation tissue as found in healing adult wounds [4]. Most importantly for this study, healing adult wounds are characterized by a marked contraction of the wound substance, thought to be mediated by tissue fibroblasts (and their cellular derivatives, myofibroblasts), whereas in fetal wounds no such contraction occurs. It has been suggested that fibroblasts/ myofibroblasts effect wound contraction either by acting together as a contractile unit, or more likely by acting individually to apply traction to a wound in the process of cell locomotion [5], [6], [7].

Multiple studies have sought to identify the key molecular agents responsible for scarless wound healing, and have examined the roles of various growth factors, cytokines, extracellular matrix (ECM) proteins [8], chaperonins and homeobox genes among others [2], [8], [9], [10], [11], [12], [13], [14], [15]. Despite observed differences in expression, however, the critical molecular mechanisms by which scarless fetal wound healing distinguishes itself from scirrhous wound healing remain unclear. The recapitulation of a fetal pattern of wound healing in adult tissues would be of enormous clinical significance, as it would allow for mitigation of scar formation and diminish the attendant morbidity.

We have previously investigated gene expression in healing fetal wounds *in vivo* with multiple expressomic techniques, including differential display [11], PCR suppression subtraction hybridization [12] and fetal wound specific microarrays (Kathju et al., manuscript in preparation). These investigations have allowed us to compare the fetal (and adult) wound expressomes without any preconceptions as to which gene products may be important. Multiple candidate genes have been found to be differentially and specifically regulated in healing fetal wounds; this report focuses on the eta subunit of the chaperonin containing T-complex polypeptide (CCT-eta), which was noted to be specifically underexpressed in fetal wounds by differential display RT-PCR [11].

The CCT molecule is the major cytosolic chaperonin in eukaryotes and has been estimated to interact with up to 15% of all cellular proteins. The structure of the CCT holoenzyme is unique among chaperonins, consisting of a pair of identical rings; each ring is composed of eight different 60-kDa subunits: alpha, beta, gamma, delta, epsilon, eta, theta and zeta [16] which are coded by eight different genes [17], [18], [19]. CCT's role as a chaperonin has been best elucidated in its interactions with the cytoskeletal proteins actin and tubulin [20], but it has also been found to assist in the folding of multiple other proteins, including cyclin E, myosin, transducin and Von Hippel Lindau tumor suppressor among others. Alteration of CCT expression thus has the potential to impose pleiotropic secondary effects on cells.

The majority of studies on CCT function have thus far focused on the holoenzyme, however, an increasing body of evidence suggests that the individual subunits of CCT have an independent function and significance. Roobol and Carden have found that not all CCT subunits co-localize identically within cells, suggesting individual subunit functionalization [21]. Genetic studies have identified several discrete CCT subunit genes as the loci for mutations resulting in neurological defects: a 143 bp deletion in CCT-gamma causes the no tectal neuron phenotype in zebrafish [22]; a missense mutation in CCT-delta causes the mutilated foot phenotype in rats [23]; a missense mutation in CCT-epsilon is responsible for mutilating sensory neuropathy with spastic paraplegia in humans [24]. Individual subunits have also been identified as the biological partners of various proteins; yeast two-hybrid analysis has identified the EBNA-3 nuclear protein from Epstein-Barr virus as binding to CCT-epsilon [25], and significantly the CCT-eta subunit has been found to serve as a co-factor for the soluble guanylyl cyclase [26].

CCT is ubiquitously expressed in all eukaryotic tissues thus far examined, and Kubota et al. describe that in mouse tissues all CCT subunits appear to be coordinately regulated [27]. Cyrne et al. [28] note that the CCT-eta and -gamma mRNAs are co-regulated during ciliary biogenesis and sexual reproduction in Tetrahymena. However, in other systems no such co-regulation is apparent. Himmelspach et al. [29] working in a plant system, find a light dependent reduction of CCT-epsilon but not CCT-alpha. Few reports examine all eight major CCT subunits.

The only examination of CCT function in wound healing thus far has centered on CCT-eta. Previous studies from our laboratory have shown the specific reduction of CCT-eta mRNA in the healing fetal wound mileu [11] and subsequently we have also shown that no other CCT subunit shares this distinct pattern of gene regulation [14]. In contrast, Koulikovska et al. [30] have shown that increased expression of CCT-eta correlated with the increased expression of alpha smooth muscle actin (α-SMA) in an adult rabbit model of corneal wounding. The substantial decrease of CCT-eta in a fetal wound healing mileu (in contrast to adult wounds) lead us to hypothesize that CCT-eta might be a critical determinant of the distinct behavior of these two phenotypes and that the regulation of CCT-eta expression might modulate the healing response of adult wounds.

We have focused our efforts on what effects specific modulation of CCT-eta levels might have on fibroblast physiology. Since fibroblasts are the ultimate effectors of scar deposition and contraction, and since wound healing (in adults) requires that they migrate into a wound bed and contract the wound substance, we have directed our studies to the examination of fibroblast motility and contractility and the role of CCT subunits therein. We first demonstrate that fetal fibroblasts express substantially less CCT-eta subunit compared to adult fibroblasts, and that they have inherently distinct characteristics of cellular locomotion and traction. Most particularly, we employ siRNAs directed against two discrete subunits (CCT-eta and CCT-beta) to demonstrate that only downregulation of the former has marked effects on the motility and contractility of adult fibroblasts, in each case shifting the adult fibroblast profile towards a more fetal-like state.

We next examined expression of cellular actin, long understood as the major cytoskeletal element in cellular locomotion and traction, and known to be a major substrate of the CCT holoenzyme. Fibroblasts are known to express two actin isoforms (namely β- and γ- actin) which are similarly expressed in all eukaryotic cell types. However, under certain conditions fibroblasts may also express the alpha-smooth muscle isoform of actin (α-SMA), eg. when stimulated by serum in tissue culture or when stimulated during adult wound healing in vivo to function as “myofibroblasts,” the derivative cell type most closely associated with wound contraction and scar formation. The presence of α-SMA has also been found to closely correlate with the appearance of scar formation even in fetal tissues that have already transitioned to the adult scar-forming phenotype in late gestation, whereas α-SMA is largely absent from earlier scarlessly healing fetal wounds [31], [32]. Thus, α-SMA expression appears to be an important differentiating feature between scarring and scarless wounds, and offers a potential mechanistic connection between CCT function, fibroblast physiology, and scar contracture.

We now report that fetal fibroblasts express less constitutive α-SMA than adult cells, and that reduction of CCT-eta markedly diminishes α-SMA protein levels, whereas reduction of CCT-beta has no such effect. Direct reduction of α-SMA leads to a similar decrease in both basal and growth-factor induced motility as seen with CCT-eta depletion, again causing adult fibroblasts to mimic a more fetal pattern of behavior.

## Materials and Methods

### Materials

Human epidermal growth factor (EGF) was obtained from Collaborative Biomedical Products (Bedford, MA). Human platelet derived growth factor (PDGF-BB) was purchased from R&D Systems (Minneapolis, MN). Antibodies against CCT-eta (cat # MCA2179) and CCT-beta (cat # MCA2275) were purchased from Serotec Inc. (Raleigh, NC). Antibody against α-SMA was purchased from Sigma Chemical Corp. (St Louis, MO). Antibody against GAPDH (cat # ab8245) was purchased from Abcam, Inc. (Cambridge, MA). RPMI-1640 medium, Trypsin-EDTA, OptiMEM and Lipofectamine 2000 were obtained from Invitrogen Corp. (Carlsbad, California). Antibiotic and antimycotic solution was purchased from Sigma Chemical Corp. Fetal bovine serum was purchased from Gemini Bio-Products (West Sacramento, CA).

### Animal Protocol and Nucleic Acid Isolation

All animal protocols were reviewed and approved by the Institutional Animal Care and Use Committee (IACUC) of the Allegheny General Hospital, Pittsburgh, PA and followed guidelines set forth in the National Institutes of Health Guide for the Care and Use of Laboratory Animals. The tissue harvesting protocol for our New Zealand white rabbits, RNA isolation/storage from fetal and adult tissues was performed as previously described [11], [12]. The quality and quantity of total RNA extracted from tissues and fibroblasts were determined by measuring the OD 260/OD 280 ratio using the ND-1000 spectrophotometer (Nanodrop Technologies Inc., Wilmington, DE) and by capillary electrophoresis with the Agilent 2100 BioAnalyzer (Agilent Technologies Inc., Palo Alto, CA).

### Dermal Fibroblast Culture

New Zealand white rabbits were anesthetized and freshly excised skin specimens (around 1 sq cm) from adult and fetal rabbits (gestational age 21 days) were minced into small pieces within 30 minutes after dissection. These tissue pieces were washed exclusively in PBS containing 1% antibiotic/antimycotic solution (Sigma, St Louis, MO) and then placed in the RPMI 1640 medium containing 10% fetal bovine serum (FBS) and 1% antibiotic/antimycotic solution. The cultures were left undisturbed for a week at 37C with 5% CO_2_ supplement. The outgrowing fibroblasts seen after a week from these primary cultures were sub-cultured immediately using 0.5% Trypsin-EDTA (Invitrogen Corp.)

### Construction of siRNAs and Transfections

Short interfering RNA (siRNA) duplexes were synthesized and purified by Integrated DNA Technologies (Coralville, IA). The siRNA sequences targeting rabbit CCT-eta, CCT-beta and alpha-SMA are shown in Table 1. A “scrambled” siRNA with an identical base pair composition but randomized sequence was also obtained as control for both the CCT-eta and CCT-beta subunits and is also shown in Table 1. A non-specific control siRNA from Abcam Inc. (Cambridge, MA) was used during α-SMA siRNA transfection. These siRNAs were employed in adult fibroblasts in transient transfection experiments wherein a) fibroblast mobility was assayed in an *in vitro* wounding protocol, and b) cellular contractility was assayed through traction force microscopy.

**Table 1:** List of siRNA sequences used to target the genes of interest.

Gene	Sequences
Rabbit CCT-eta	Sense-5′ rGrArArCrGrArUrUrCrArGrUrArGrUrGrGrCrUTT 3′Antisense-5′ rArGrCrCrArCrUrArCrUrGrArArUrCrGrUrUrCTT 3′
Rabbit CCT-beta	Sense-5′rGrGrArGrArArArGrUrUrGrArArCrGrUrArUrUTT-3′Antisense-5′rArArUrArCrGrUrUrCrArArCrUrUrUrCrUrCrCTT3′
Rabbit α-SMA	Sense- 5′rArGrArGrArArArUrUrGrUrGrCrUrArUrGrUrCTT3′Antisense-5′rGrArCrArUrArGrCrArCrArArUrUrUrCrUrCrUTT3′
Scramble Control CCT-eta	Sense-5′rGrArArcrGrArUrUrCrGrArArUrGrCrUrGrGrUTT3′Antisense-5′rArCrCrArGrCrArUrUrCrGrArArUrCrGrUrUrCTT3′
Scramble Control CCT-beta	Sense-5′rUrGrArArGrArArGrGrArArGrUrUrGrCrUrArUTT3′Antisense-5′rArUrArGrCrArArCrUrUrCrCrUrUrCrUrUrCrATT3′

Rabbit adult fibroblasts were cultured in RPMI 1640 supplemented with 10% fetal bovine serum. Transfection of siRNAs was performed with the manufacturer's protocol using Lipofectamine 2000. Briefly, 7.5 µl of 20 µM siRNA was mixed with 200 µl of Opti-MEM; 4 µl of Lipofectamine 2000 was diluted into 200 µl of Opti-MEM and incubated at room temperature for 5 min. After the incubation, the diluted Lipofectamine 2000 was combined with the diluted siRNAs and then incubated for an additional 20 min (siRNA sequences targeting both CCT subunits and α-SMA were used at a concentration of 150 pM). A total of 400 µl of siRNA-Lipofectamine 2000 complexes was added to each well of cultured rabbit adult fibroblasts at ≈90% confluence in a six well plate. After 24 h incubation at 37C the cells were switched to quiescent media (RPMI 1640 medium containing 0.1% dialyzed FBS along with antibiotics) and left for 48 h. After 48 h of incubation in quiescent media cells were subjected to the *in vitro* wounding protocol described below; at this same time, cell populations were also stimulated either with EGF (1 nM)/ PDGF (200 nM) or control. Thus, during the period of cell motility assayed these growth factors (or control, that is, no treatment) were continuously present. To validate the sequence-specificity of gene knock-down *in vitro*, after transfection the fibroblasts were analyzed for protein and mRNA downregulation by immunoblots and real-time RT-PCR.

### Quantitative Real Time RT-PCR

Total RNA isolated (RNeasy Micro Kit, Qiagen Inc., Valencia, CA) from rabbit adult fibroblasts after CCT-eta , CCT-beta and α-SMA siRNA transfection was subjected to real time RT-PCR to determine the abundance of these two CCT subunit messages. Primers and probes for these assays were designed by Primer Express software (Applied Biosystems, Foster City, CA) as described previously (Satish et al., 2010 manuscript submitted). The primers were obtained from Integrated DNA Technologies and the Taqman probes were purchased from Applied Biosystems. The experimental conditions for RT-PCR were carried out exactly as described previously [14]. Using the comparative critical cycle (C_t_) method and using GAPDH as the endogenous control, the expression levels of the target gene products were normalized and the relative abundance was calculated. Data were analyzed using the 7900 HT SDS software version 2.1 provided by Applied Biosystems.

Total RNA extracted from rabbit adult and fetal fibroblasts was similarly subjected to these quantitative comparative RT-PCR assays to determine the relative mRNA expression levels of CCT-eta, CCT-beta and α-SMA in these cell types at baseline.

### Immunoblotting

Rabbit adult fibroblasts were lysed using SDS-sample buffer and proteins were analyzed after siRNA transfection against CCT-eta and CCT-beta. Proteins from equal volumes of cytosol from the various experimental conditions were resolved by SDS-PAGE and analyzed by immunoblotting with antibodies specific for CCT- eta, CCT-beta, and α-SMA. Antibody against GAPDH was used as a loading control. Equal amounts of protein lysates isolated from non-transfected control rabbit adult and fetal fibroblasts were also subjected to SDS-PAGE and probed with antibodies specific for CCT-eta, CCT-beta and α-SMA with GAPDH serving as a loading control.

### Cell Migration Assay

Rabbit adult fibroblasts were plated on 6-well plastic dishes and grown to confluence in RPMI 1640 supplemented with 10% FBS. The cells were quiesced for 48 h in the media containing 0.1% dialyzed FBS; a switch in the media reduces cellular proliferation and thymidine incorporation over 90% while maintaining cell viability [33], [34]. An *in vitro* wound healing assay was performed as previously described [35]. In brief, near confluent cultures of rabbit adult fibroblasts were scraped making an ∼1-cm wide denuded area, then either stimulated with cytokines {EGF (1 nM) or PDGF (200 nM)} or with control vehicle only; photographs were taken at 0 h and 48 h and the relative distance traveled by the cells at the acellular front was determined by computer-assisted image analysis. Markings on the plates ensured measurement at the same site of the photographs at each time point. The distance migrated was then expressed as a percentage of the control distance migrated within each experiment, which also facilitated inter-experiment comparisons.

### Measuring Traction of Individual Rabbit Adult Skin-derived Fibroblasts

Traction, the cellular force that an individual adherent cell exerts on its substrate, was determined using traction force microscopy [36]. In this technique, scattered fibroblasts are plated on a collagen matrix into which are embedded fluorescently-labelled microbeads. Fibroblast cells attach to the matrix and, in keeping with their physiology, begin to apply a traction force to it, resulting in micro-displacements of the embedded beads. The quantity of displacement is measurable and can be analyzed through computer-assisted algorithms. Fibroblast populations treated with multiple agents can be compared by analyzing a sufficient number of cells from each experimental condition.

Briefly, a polyacrylamide gel disk (120 µm thick, 10 mm in diameter) embedded with 0.2 µm green fluorescent micro-beads (Molecular Probes, Eugene, OR) was made and attached to the bottom of a 35 mm glass dish (with a 14 mm circular inner glass area) (MatTek, Ashland, MA) that was consecutively pretreated with 0.1M sodium hydroxide, 3-aminopropyltrimethoxysilane, and 0.5% glutaraldehyde. The gel surface was then pretreated with Sulfo-Sanpah (Pierce, Rockford, IL) and coated with 200 µl of 100 µg/ml collagen type I.

Rabbit adult and fetal fibroblasts in passage 2–4 were grown in RPMI-1640 medium containing 10% fetal bovine serum (FBS) and 1% antibiotic/antimycotic solution as mentioned above. Initial experiments measured the traction force exerted by adult fibroblasts relative to that exerted by fetal fibroblasts. We next investigated the effect of growth factor treatment (using EGF and PDGF) on adult fibroblast contractility as reflected in the applied traction force. We then examined whether reduction of CCT-eta or CCT-beta via siRNA transfection would affect the contractility of fibroblast cells, either at baseline or in response to growth factor (PDGF).

For experiments involving siRNA, cells were transfected (as described above) with CCT-eta and CCT-beta siRNAs and were also co-transfected with the plasmid pdsRed2-C1 (Clontech Laboratories, Inc., Mountain View, CA). This vector contains the red fluorescent protein gene driven by a cytomegalovirus (CMV) promoter, known to be widely expressed in a variety of cell types. After 24 h transfection the cells were switched to quiescent media (RPMI 1640 medium containing 0.1% dialyzed FBS along with antibiotic/antimycotic solution) and left for another 24 h at 37C. Then, 48 h after transfection, fibroblasts were trypsinized off their tissue culture vessels and were placed on the above-described collagen-coated polyacrylamide gel disks at 3000 cells/disk +/− PDGF (200 nM) and allowed to spread on the collagen surface gel for 6 h in quiescent medium at 37C. Cells visibly expressing red fluorescence after this time were selected (at least 5 cells per disk); these cells are proven to be successfully transfected with pdsRed2-C1, and are therefore highly likely to have taken up siRNA as well. This allowed us to collect traction force measurements only from cells that were likely exhibiting an siRNA mediated downregulation of the CCT-eta/CCT-beta subunits, rather than relying on capturing a sufficient number of cells by surveying a large group, which would necessarily include many non-transfected cells. First, phase contrast images of individual cells as well as red fluorescent images were taken, followed by imaging of the underlying green fluorescent beads. Then, the cells on the gel disk were trypsinized (removing the applied contractile force) and images of the fluorescent beads at the same view and the same z plane were taken.

An image correlation method was used to compute the bead displacement field by comparing the bead image under the contracting cells with the cell-free bead image. At the beginning of the image processing, the pair of images was corrected for relative translational shift. Then the images were divided into small windows (16 by 16 pixels), and the normalized cross-correlation function was implemented for each window to find the relative displacement for each window between these two images. A hierarchical algorithm, polynomial fitting, and low-pass filtering was used to minimize the noise level. The traction field was calculated from the displacement field by using Fourier transform traction cytometry (FTTC) [36]. The calculation of traction forces is based on the Boussinesq solution for the displacement field on a surface of a semi-infinite solid [37]. At least 20 cells in each experimental group were used to determine their collective average traction forces.

### Statistical Analyses

Statistical analyses were performed by a paired two-tailed Student's *t*-test. A p value of <0.05 was considered significant.

## Results

### CCT-eta mRNA and protein levels are reduced in fetal fibroblasts when compared to adult fibroblasts

**Figure 1:**
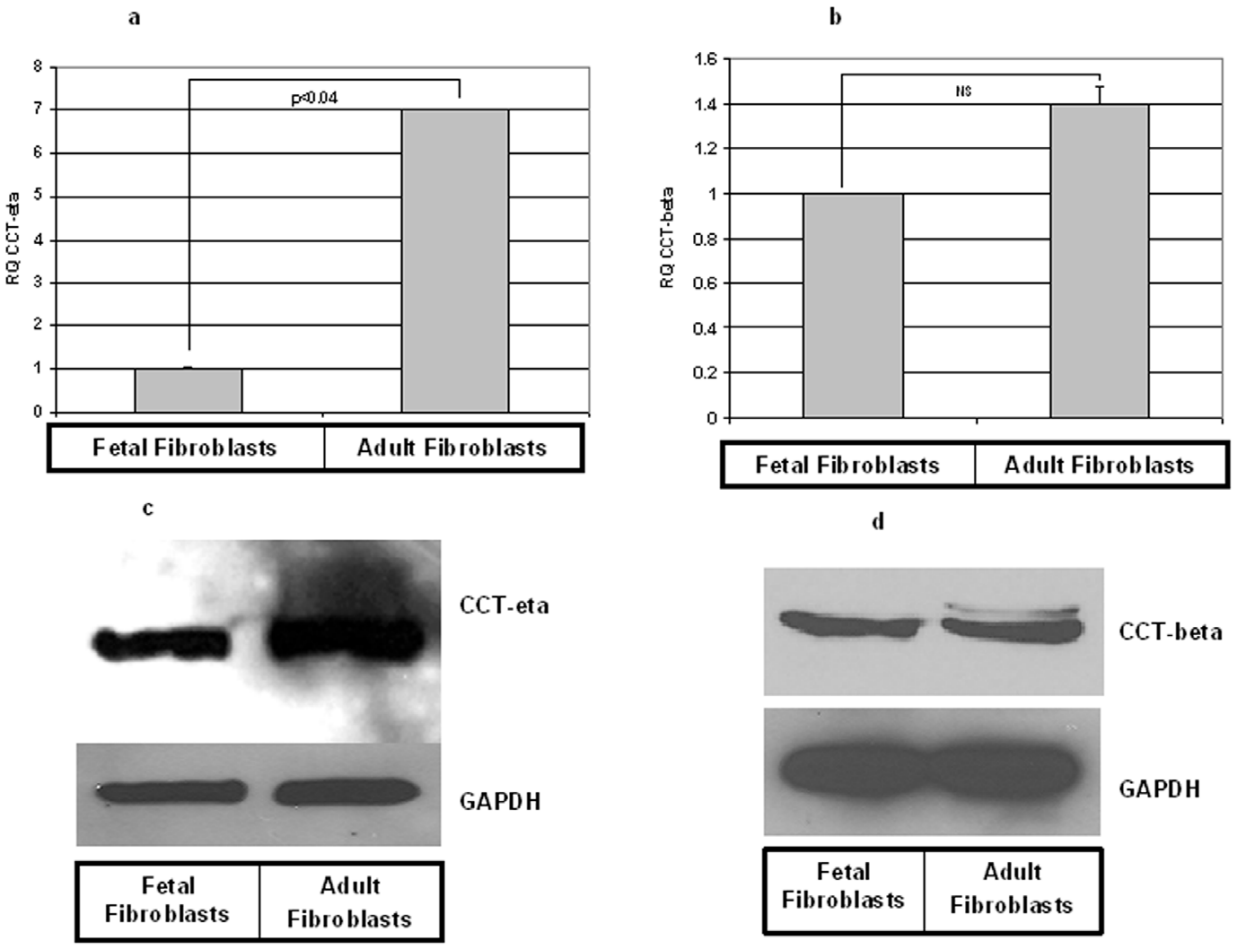
CCT-eta but not CCT-beta protein and mRNA are differentially expressed in fetal versus adult fibroblasts. RNA and protein extracted from fetal and adult fibroblasts were subjected to qRT PCR (a & b) and Western blot (c & d) analyses respectively. CCT-eta mRNA was significantly more abundant in adult fibroblasts when compared to fetal fibroblasts (a); there was no significant difference in CCT-beta message levels between fetal and adult fibroblasts (b). Values are means ± SEM of three independent studies performed in duplicate. Statistical analyses were performed using Student's *t* test. NS = non-significant. Equal amounts of protein loaded from fetal and adult fibroblasts showed that adult fibroblasts express significantly greater CCT-eta protein(c). In contrast CCT-beta protein levels were not different between fetal and adult fibroblasts (d). Blots shown here are representative of at least three different experiments.

We first examined both CCT-eta and CCT-beta message and protein levels in primary cultures of fetal and adult fibroblasts. qRT-PCR indicated that CCT-eta mRNA expression was significantly elevated in adult fibroblasts (by ∼ seven- fold) when compared to fetal fibroblasts (Figure 1a); in contrast, there was little difference in CCT-beta messenger abundance between fetal and adult fibroblasts (Figure 1b). Immunoblotting demonstrated that CCT-eta protein expression was similarly substantially elevated in adult fibroblasts compared to fetal fibroblasts, whereas CCT-beta protein expression showed no difference between the two cell types (Figures 1c and 1d).

### Cell migration of fetal and adult fibroblasts is differentially modulated by exogenous growth factors

**Figure 2:**
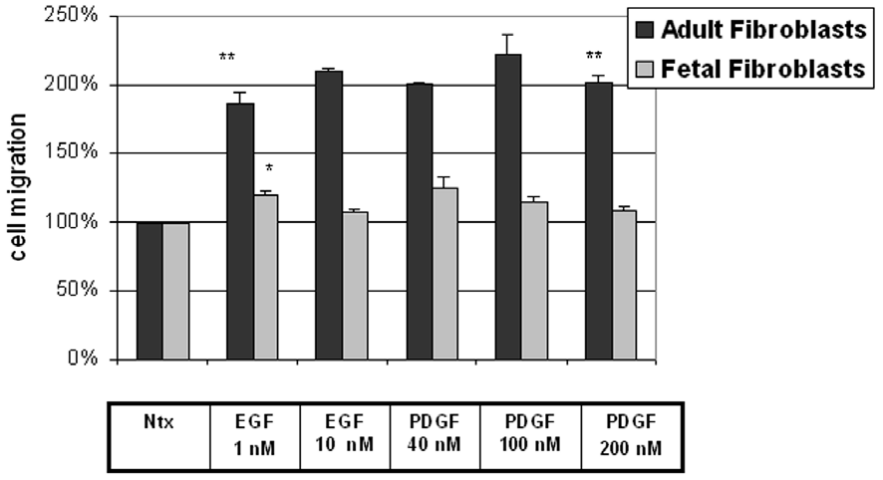
Cell migration of adult but not fetal fibroblasts is responsive to EGF and PDGF induction. Primary cultures of fibroblasts obtained from fetal and adult rabbit skin were tested for motility in an *in vitro* wound healing assay. Cells were treated with increasing concentrations of EGF (1 nM and 10 nM) and PDGF (40 nM, 100 nM, 200 nM). The values are normalized to baseline motility and shown as EGF- and PDGF-induced cell motility at each concentration. Fetal and adult fibroblasts had essentially identical baseline motility, but only adult cells responded to growth factor stimulation. The values are mean ± SEM of six independent studies each performed in triplicate. Statistical analyses were performed by Student's *t*-test.

Since one of the main phenotypic attributes of fibroblastic cells relevant to their role in wound healing and scar formation is their ability to migrate into a wound bed, we investigated the migration profiles of fetal and adult fibroblasts in a well-established *in vitro* assay [35], both at baseline and in response to several growth factors known to be important in the adult wound healing process (EGF and PDGF). Fetal and adult fibroblasts were noted to have comparable migratory profiles at baseline, but, as shown in Figure 2, fetal fibroblasts were completely insensitive to the stimulation of cell migration seen in adult fibroblasts in response to EGF and PDGF. Whereas adult fibroblasts significantly increased their migration rates in response to several concentrations of these growth factors, fetal fibroblast migration was unaffected. Although many cellular and molecular components are involved in cell migration, this striking observation led us to query whether selective reduction of the CCT-eta subunit alone might alter the migratory abilities of adult fibroblasts, in effect rendering them more “fetal-like” in behavior.

### siRNA against CCT-eta and CCT-beta effectively and specifically downregulates target mRNA and protein in rabbit adult fibroblasts

We employed siRNA technology to experimentally decrease the amount of CCT-eta and CCT-beta message (and protein) in cultures of adult fibroblasts. Cells were transfected either with experimental siRNAs (against actual target sequence) or with scrambled control siRNAs (with identical base pair compositions). After 48 h transfected cells were harvested and analyzed by qRT-PCR and immunoblotting.

**Figure 3:**
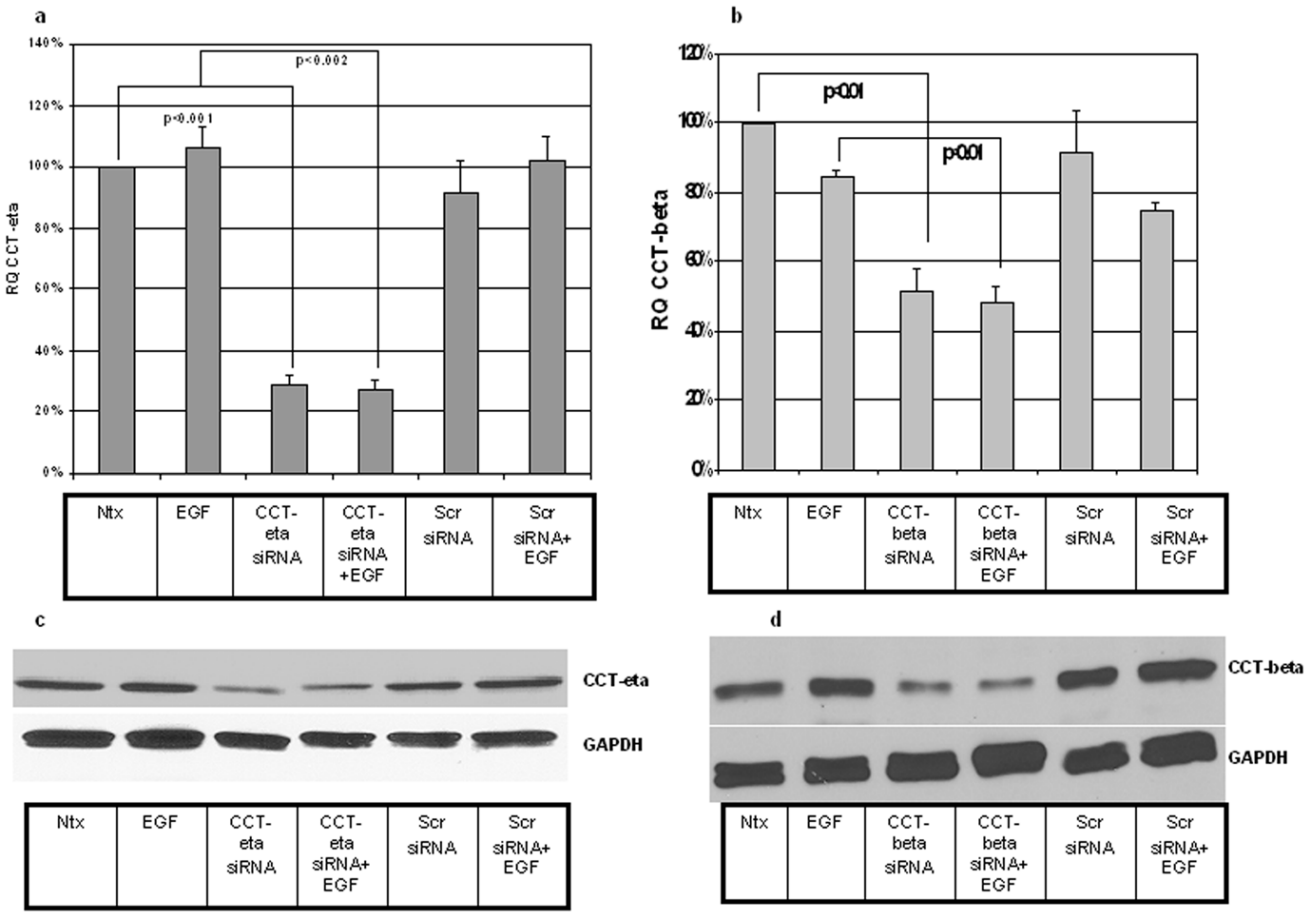
siRNAs against CCT-eta and CCT-beta decrease both basal and EGF- induced mRNA and protein levels of their targets in fibroblasts. (a & b) qRT-PCR analysis of CCT-eta and CCT-beta mRNA levels showed effective inhibition of both basal expression and EGF-induction in siRNA-transfected adult fibroblasts. Results are expressed as relative quotient (RQ) of measured CCT-eta or CCT-beta mRNA and were calculated as a percentage of baseline control levels (100%). Values are means ± SEM of six independent studies, each performed in duplicate. Statistical analyses were performed with Student's *t* test. Ntx- no transfection; EGF-EGF treatment (1 nM); siRNA-treatment with CCT-eta/CCT-beta siRNA; Scr- treatment with scrambled control siRNA. (c & d) Western blot results using CCT-eta and CCT-beta antibody (1∶500) showed effective reduction of CCT-eta and CCT-beta protein levels when siRNA was administered but no decrease when scrambled siRNA was employed. GAPDH was used as a loading control. A representative immunoblot of up to four similar such blots is shown for each analysis.

siRNA versus CCT-eta reduced mRNA levels to 29% of control; this reduction was effective even in the presence of EGF (Figure 3a) (similar results were obtained with PDGF- data not shown). Scrambled siRNA sequence had no effect on CCT-eta message. Neither CCT-beta nor CCT-alpha message levels were affected by siRNA targeted against CCT-eta (data not shown). Importantly, CCT-eta siRNA also significantly reduced CCT-eta protein levels as measured by Western blot (Figure 3c); again, this reduction was effective even in the presence of added growth factors, and control scrambled siRNA elicited no such decrease in target protein.

siRNA targeted against CCT-beta was similarly effective, reducing CCT-beta message by some 50% (by qRT-PCR) and CCT-beta protein by greater than 50% (by Western blot) (Figures 3b and 3d). As with CCT-eta, this reduction held even in the presence of growth factors, and control scrambled siRNA sequence had no effect on either mRNA or protein levels. CCT-beta siRNA had no effect on CCT-eta or CCT-alpha message levels (data not shown).

### siRNA against CCT-eta but not CCT-beta inhibits EGF- and PDGF-induced cell migration in adult fibroblasts

Since we have confirmed that CCT-eta is specifically decreased during fetal wound healing, and that CCT-beta is not differentially expressed in either fetal or adult wounds, we were interested to explore the functional significance of the specific inhibition of these target genes, CCT-eta and CCT-beta, on cellular characteristics important to wound healing and scar formation. We therefore investigated what effect the downregulation of CCT-eta and CCT-beta by siRNA in adult fibroblasts would have on their ability to migrate in an *in vitro* wound healing assay. Near confluent cultures of adult fibroblasts were transfected with siRNA versus CCT-eta (or CCT-beta) or control, then a zone of “wounding” was created by mechanically removing cells with a rubber policeman. Cultures were then stimulated with growth factors (or control vehicle only) and incubated for 48 h while cells moved to repopulate the denuded “zones of injury.” The distance the cells migrated from the acellular front edge was photographed and measured with computer assistance, thus arriving at a quantitative value reflecting cellular motility.

**Figure 4:**
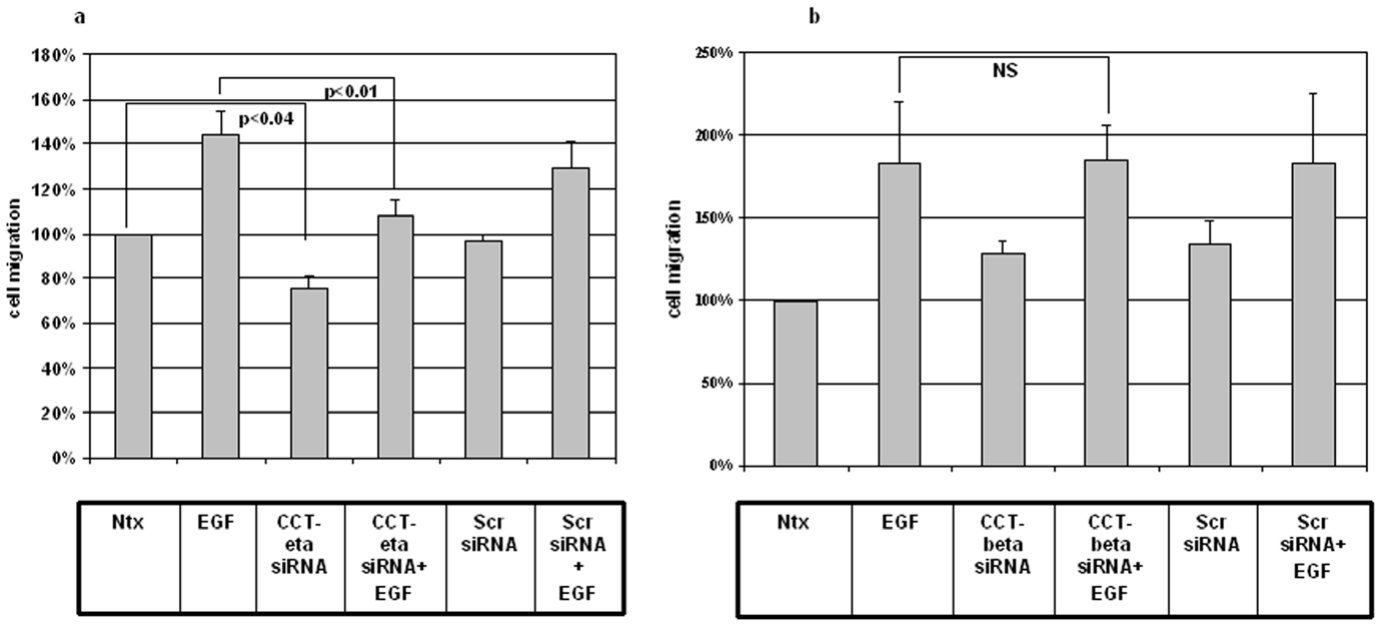
siRNA against CCT-eta decreases EGF - induced fibroblast migration, whereas siRNA against CCT-beta does not. Cells were incubated in the presence or absence of EGF(1 nM) +/− siRNA against CCT-eta (a) or CCT-beta (b) in an *in vitro* wound healing assay. In all experiments a subunit -specific scrambled siRNA sequence was used as control. Cell motility is displayed as a relative percentage of baseline motility in the absence of EGF or siRNA exposure (100%). Active siRNA versus CCT-eta reduced both basal and EGF-induced motility; siRNA versus CCT-beta and scrambled controls had no effect. Values are means ± SEM of six independent studies, each performed in triplicate. Statistical analyses were performed with Student's *t* test.

The results of this *in vitro* wound healing assay, which measures the averaged and directional migration of a cell population, were striking and consistent: siRNA-mediated reduction of CCT-eta markedly decreased basal cell motility to 76%±6% (p<0.04) compared to no treatment and decreased epidermal growth factor (EGF)-induced cell motility to 108%±8% (p<0.01) compared to EGF treatment alone (144%±11%, p<0.02) (Figure 4a). In contrast downregulation of CCT-beta with siRNA did not result in any inhibition of either basal cell motility or EGF-induced cell motility (Figure 4b). In no case did scrambled siRNA have any effect on cellular migration when compared to untreated controls.

**Figure 5:**
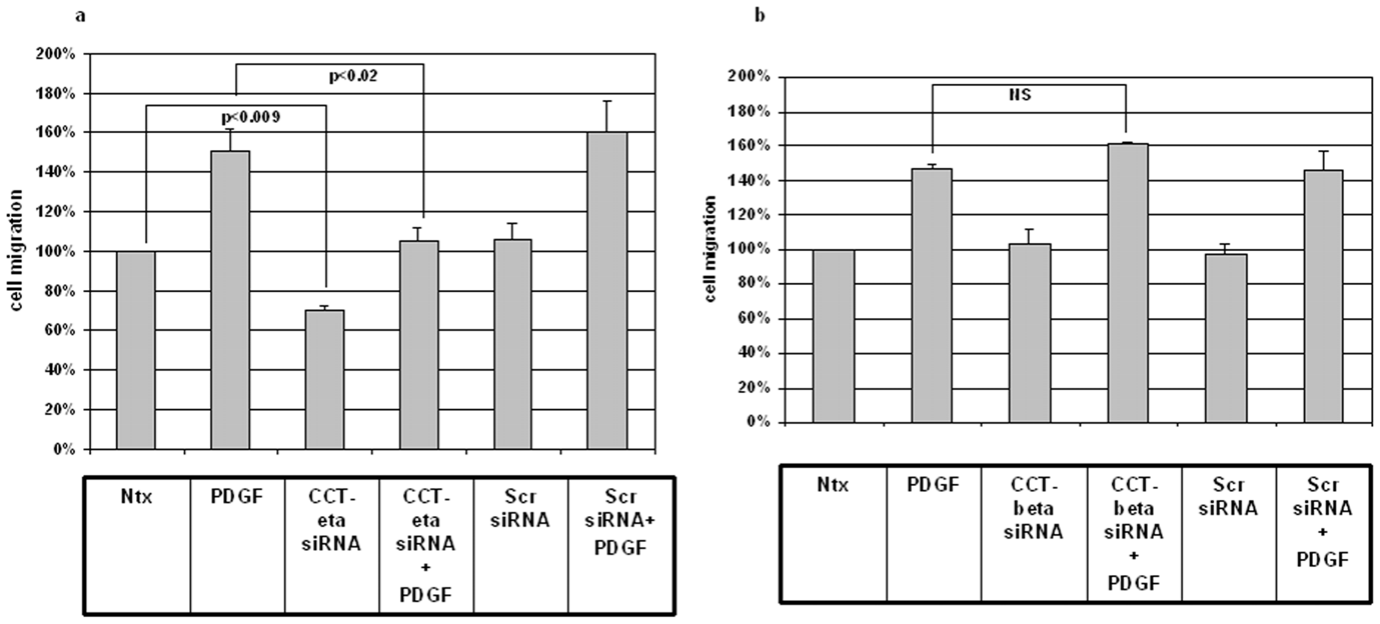
siRNA against CCT-eta decreases PDGF-induced fibroblast migration, whereas siRNA against CCT-beta does not. Cells were incubated in the presence or absence of PDGF (200 nM) +/− siRNA against CCT-eta (a) or CCT-beta (b) in an *in vitro* wound healing assay. In all experiments a subunit -specific scrambled siRNA sequence was used as control. Cell motility is shown as a percentage of baseline migration in the absence of PDGF or siRNA exposure. As with EGF, active siRNA targeting CCT-eta inhibited basal and PDGF-induced motility, whereas CCT-beta siRNA and scrambled controls did not. Values are means ± SEM of six independent studies, each performed in triplicate. Statistical analyses were performed with Student's *t* test.

Virtually identical results were obtained when examining the effect of CCT subunit downregulation on cell migration in response to PDGF. Once again downregulation of CCT-eta inhibited both basal cell motility (70%±2%, p<0.009 compared to no treatment) and PDGF-induced cell migration (to 105%±7%, p<0.01) compared to PDGF treatment alone (151%±11%, p<0.05). As in our experiments with EGF, downregulation of CCT-beta did not have any effect on either basal or PDGF-induced cell migration (Figures 5a & 5b).

In each of these experiments we confirmed the successful donwregulation of CCT-eta (or CCT-beta) mRNA and protein levels by qRT-PCR and Western blot (as presented in Figure 3) (data not shown). This evidence strongly supports an important and specific role for CCT-eta in fibroblast physiology and reinforces its likely importance to wound healing.

### Traction force exerted by adult fibroblasts is higher than fetal fibroblasts

**Figure 6:**
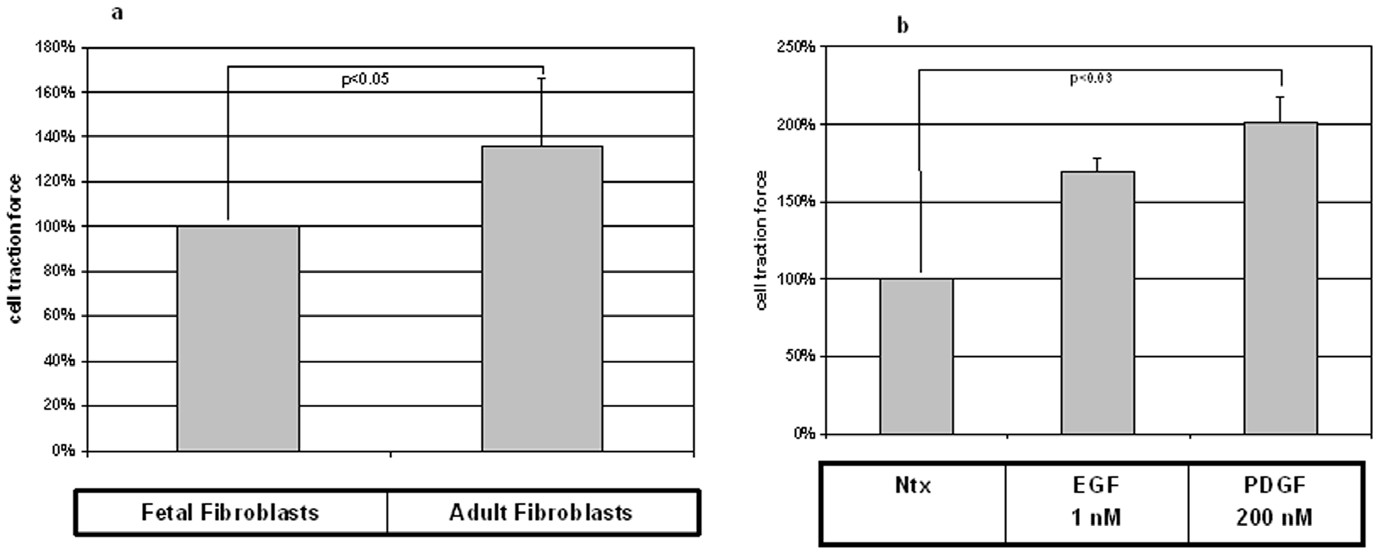
Adult fibroblasts are more contractile than fetal fibroblasts. (a) Fetal fibroblasts are less contractile than adult fibroblasts as determined by traction force microscopy. Each bar represents mean ± SEM of more than 20 cells from two independent experiments. Statistical analyses were performed using Student's *t* test. (b) PDGF treatment of adult fibroblasts results in an increase in the observed cumulative traction force; EGF treatment results in a similar although smaller increase. Each bar represents mean ± SEM of more than 25 cells from two different experiments. Statistical analyses were performed using Student's *t* test.

Having observed that modulation of CCT-eta can affect fibroblast motility, we also sought to measure its effect on cellular contractility. Since one of the major differentiating characteristics of fetal versus adult wound repair is that the latter proceeds with a contraction of the wound bed, we hypothesized that fetal fibroblasts may be inherently less contractile than adult. We therefore first compared the traction forces exerted on their substrata by fetal fibroblasts versus adult fibroblasts using traction force microscopy. Under basal conditions adult fibroblasts apply a significantly stronger traction force (136%±29% p<0.04) to their microenvironment than do fetal fibroblasts (Figure 6a).

### Adult fibroblast contractility is enhanced by stimulation with growth factors

We next investigated what effect EGF and PDGF have on adult fibroblast contractility. Cultures of adult fibroblasts were incubated with these growth factors (or control vehicle) and the ability of their component cells to exert traction on their substrata was again quantified by traction force microscopy. Interestingly, although both agents markedly increased cellular contractility, we found that PDGF stimulated cellular traction force (201%±15% p<0.03) more highly than EGF (169%±9%) (Figure 6b). Since traction force microscopy is a highly labor intensive assay, this observation led us to select PDGF as the stimulator to use in further traction force experimentation.

### siRNA against CCT-eta but not CCT-beta inhibits PDGF-induced traction force in adult fibroblasts

**Figure 7:**
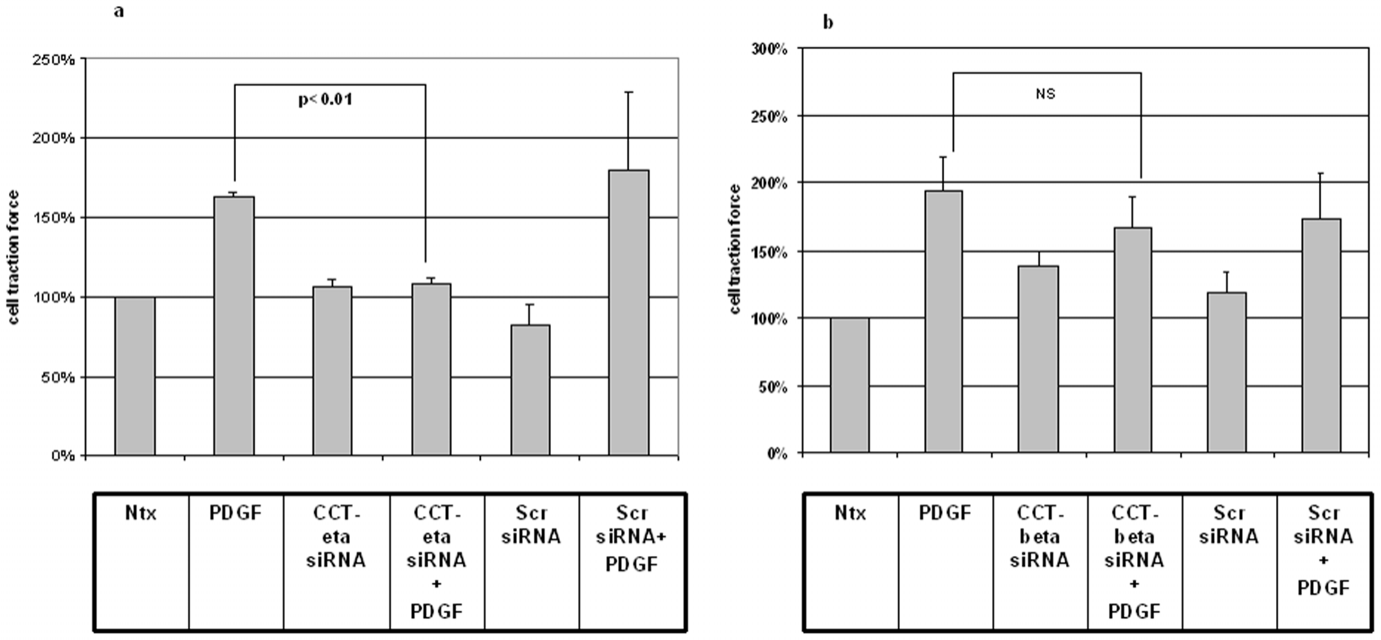
siRNA against CCT-eta but not CCT-beta reduces PDGF-induced cellular traction force in adult fibroblasts. Adult fibroblasts transfected with CCT-eta (a) or CCT-beta siRNA (b) along with pDSRed2-C1 were quantified for microdisplacement fields of red fluorescent cells on the green fluorescent substrate. Each assay was repeated twice with more than 30 cells quantified in each experiment. CCT-eta siRNA abolished the increased cellular traction force seen with PDGF treatment (200 nM), whereas CCT-beta siRNA and scrambled controls did not. Values are means ± SEM of two independent experiments with statistical analyses performed using Student's *t* test.

We next examined the effect of downregulation of CCT-eta by siRNA treatment on the cellular contractility of adult fibroblasts. Cultures of adult fibroblasts were transfected as described above with CCT-eta/CCT-beta siRNA (or the relevant controls), then stimulated with PDGF treatment. Cultures were simultaneously transfected with a marker vector expressing red fluorescent protein; this allowed us to selectively assay the traction forces of cells known to have taken up exogenous genetic material, maximizing the likelihood that such cells had also incorporated our siRNAs of interest (when present). Our results demonstrate that CCT-eta siRNA, while having no effect on the contractility of unstimulated cells, does abrogate the increased traction/contractility seen with PDGF treatment (108%±4%, p<0.01) compared to PDGF treatment alone (163%±3%, p<0.02). In contrast, CCT-beta siRNA has no effect on either basal cell contractility or on the PDGF-dependent increase in cellular traction (Figures 7a and 7b). Scrambled siRNAs had no effect on cellular traction in any instance. Our data therefore indicate that, in addition to diminishing the motile response of fibroblast cells, selective downregulation of CCT-eta can also inhibit their ability to contract in response to exogenous agents. This reduced potential contractility may also play a role in discriminating between a fetal and adult wound healing phenotype.

### α-SMA levels are significantly elevated in adult fibroblasts in comparison to fetal fibroblasts

**Figure 8:**
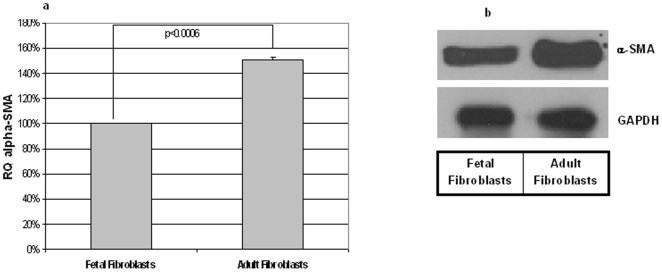
mRNA and protein levels show that α-SMA level is significantly increased in adult fibroblasts in comparison to fetal fibroblasts. RNA and protein extracted from fetal and adult fibroblasts were subjected to qRT PCR (a) and Western blot (b) analyses respectively. The α-SMA mRNA levels were significantly more abundant in adult fibroblasts when compared to fetal fibroblasts (a). Values are means ± SEM of three independent studies performed in duplicate. Statistical analyses were performed using Student's *t* test. NS = non-significant. Equal amounts of protein loaded from fetal and adult fibroblasts showed that adult fibroblasts express significantly greater α-SMA protein (b). GAPDH was used as loading control.

Since chaperonins are known to mediate the folding of cytoskeletal actin, as an initial study we determined the mRNA and protein levels of α-SMA in fetal and adult fibroblasts. We found that both mRNA and protein levels of α-SMA were significantly higher in adult fibroblasts when compared to fetal fibroblasts (Figures 8a and 8b).

### siRNA against CCT-eta but not CCT-beta markedly reduces α-SMA protein levels but did not affect beta-actin levels in adult fibroblasts

**Figure 9:**
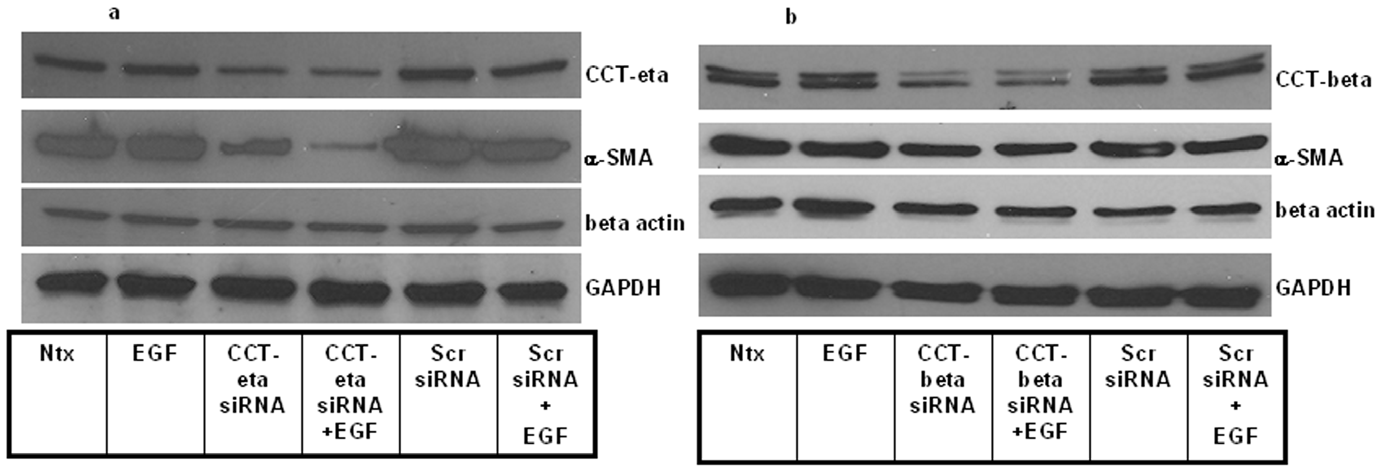
siRNA downregulation of CCT-eta but not CCT-beta reduces α-SMA protein levels in adult fibroblasts. (a & b)Western blot showed effective reduction of both CCT-eta and α-SMA protein levels when CCT-eta siRNA was administered, leaving beta-actin largely unaffected. In contrast, downregulating CCT-beta did not lead to significant reduction in either α-SMA or beta-actin levels. GAPDH was used as loading internal control. A representative immunoblot of up to three similar such blots are shown for each analysis. Ntx- no transfection; EGF-EGF treatment (1 nM); siRNA-treatment with CCT-eta/CCT-beta siRNA; Scr- treatment with scrambled control siRNA.

We next sought to determine what effect inhibiting CCT-eta and –beta subunits specifically would have on cellular α-SMA expression. We found that inhibiting CCT-eta resulted in a dramatic and specific decrease in α-SMA protein but did not have any effect on cellular beta-actin protein (Figure 9a). In contrast, CCT-beta inhibition showed no significant effect on either α-SMA or beta-actin protein accumulation (Figure 9b).

### siRNA against α-SMA effectively and specifically downregulates target mRNA and protein in rabbit adult fibroblasts

We next wished to examine the effect of direct downregulation of α-SMA on basal and EGF-induced cell migration. To this end we first synthesized an siRNA specifically targeting rabbit α-SMA. Adult fibroblasts were transfected either with experimental siRNA (against actual target sequence) or with a non-specific control siRNA. After 48 h the transfected cells were harvested and analyzed by qRT-PCR and immunoblotting.

**Figure 10:**
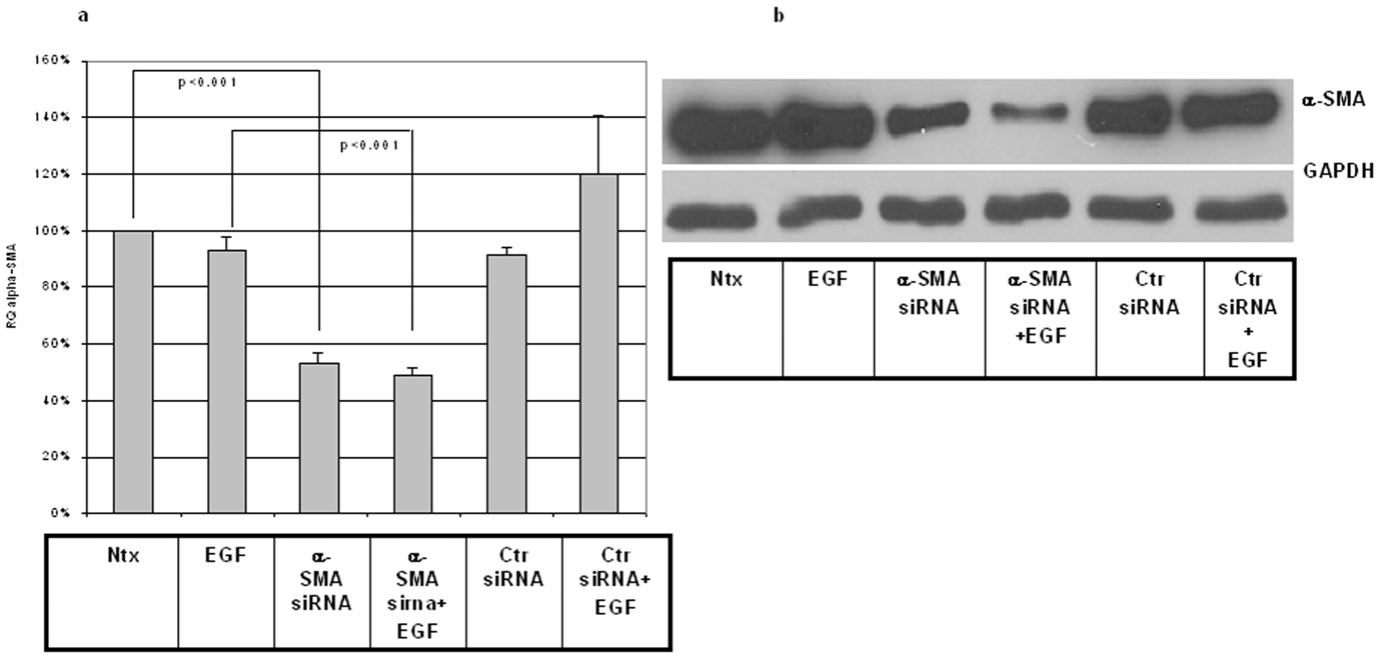
siRNA against α-SMA specifically decreases both both basal and EGF- induced mRNA and protein levels of α-SMA in adult fibroblasts. (a) qRT-PCR analysis of α-SMA mRNA levels showed effective inhibition of both basal expression and EGF-induction in siRNA-transfected adult fibroblasts. Results are expressed as relative quotient (RQ) of measured α-SMA mRNA and were calculated as a percentage of baseline control levels (100%). Values are means ± SEM of six independent studies, each performed in duplicate. Statistical analyses were performed with Student's *t* test. Ntx- no transfection; EGF-EGF treatment (1 nM); siRNA-treatment with α-SMA siRNA; Ctr- treatment with a non-specific control siRNA. (b) Western blot results using α-SMA antibody (1∶500) showed effective reduction of α-SMA protein levels when siRNA was administered but no decrease when non-specific control siRNA was employed. GAPDH was used as a loading control. A representative immunoblot of up to four similar such blots is shown for each analysis.

siRNA versus α-SMA reduced mRNA levels to ∼50% of control; this reduction was effective even in the presence of EGF (Figure 10a). The non-specific siRNA control had no effect on α-SMA message. Importantly, α-SMA siRNA also significantly reduced α-SMA protein levels as measured by Western blot (Figure 10b); again, this reduction was effective even in the presence of added growth factor, and non-specific siRNA control elicited no such decrease in target protein. No effect on cellular beta-actin was seen with either siRNA construct (data not shown).

### siRNA against α-SMA inhibits both basal and EGF-induced cell migration in adult fibroblasts

**Figure 11:**
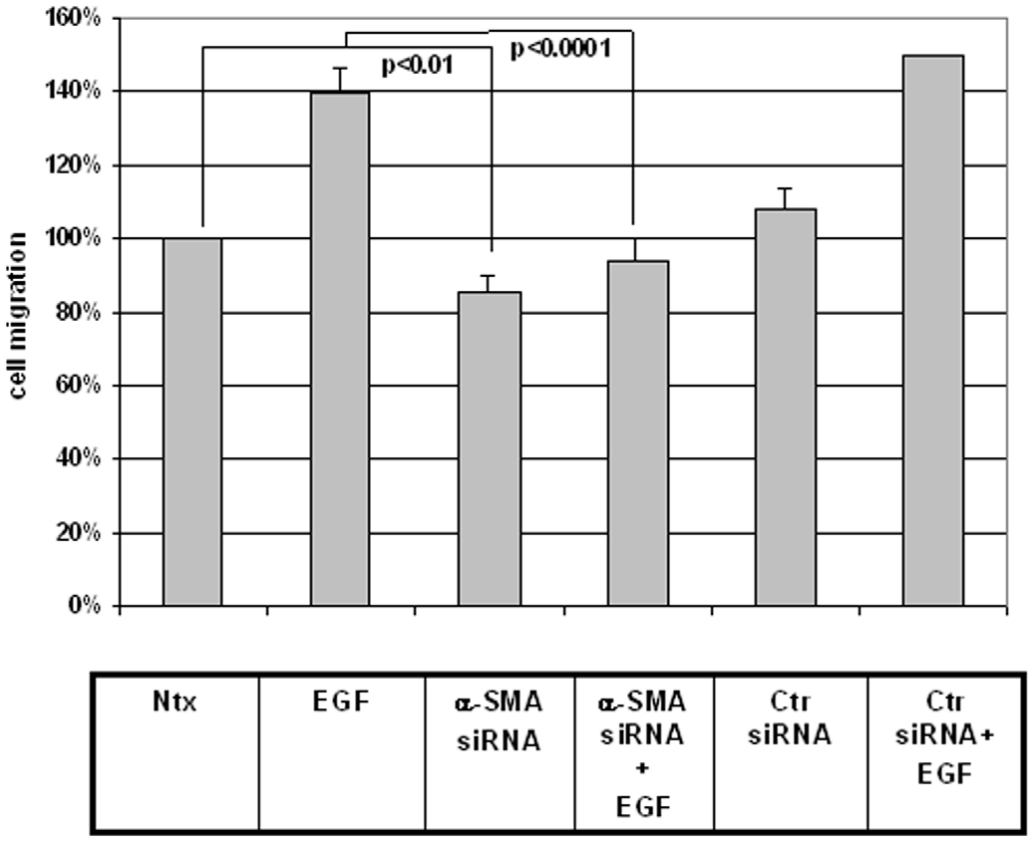
siRNA against α-SMA inhibits both basal and EGF-induced cell migration in adult fibroblasts. Cells were incubated in the presence or absence of EGF(1 nM) +/− siRNA against α-SMA in an *in vitro* wound healing assay. In all experiments a non-specific control siRNA was used as a control. Cell motility is displayed as a relative percentage of baseline motility in the absence of EGF or siRNA exposure (100%). Active siRNA versus α-SMA reduced both basal and EGF-induced motility; a non-specific control siRNA had no such effect. Values are means ± SEM of eight independent studies, each performed in duplicate. Statistical analyses were performed with Student's *t* test.

Direct reduction of α-SMA was then accomplished using siRNA against α-SMA in the setting of our *in vitro* wound healing assay to determine what effect this would have on fibroblast motility. As seen in Figure 11, depletion of α-SMA resulted in both decreased basal cell motility (86%±6% p<0.01 compared to no treatment) and in an abrogation of the ability of the cell to respond to the EGF motive stimulus (to 94%±4% p<0.001). Control siRNA had no such effects. These results mirror the phenotypic changes effected by the specific depletion of the CCT-eta subunit.

## Discussion

Although the scarless wound healing observed in fetal integumentary tissues has been known for some time, the critical mechanisms that enable tissue repair to occur absent scar formation are still unclear. Since fibroblasts are the end effector cells of scar formation, ultimately such mechanisms must manifest themselves in fibroblast physiology. This report examines two parameters of fibroblast physiology critical to wound healing and scar formation, motility and contractility, and how they are affected by modulation of the CCT subunits eta and beta.

cDNAs of CCT-eta and –beta from rabbit confirm that these polypeptides display a significant degree of sequence homology, confirming previous analyses that these subunits (and indeed, all CCT subunits) likely derive from a common ancestor gene (Satish et al., submitted). Of the eight subunits that comprise the CCT holoenzyme, however, only CCT-eta was found to be reduced in healing fetal wounds, suggesting that it might play a unique role in the physiology pertinent thereto. Since CCT-beta is the subunit most closely evolutionarily related to –eta, and since –beta displayed no evident change in expression in healing fetal (or adult) wound tissues, we have investigated CCT-beta as the most appropriate “control” subunit for –eta and its potentially unique importance to fibroblast behavior.

The reduction of CCT-eta in a healing fetal wound is apparent as early as 12 hours post-wounding. A wound milieu has multiple cell types within it, and it is as yet uncertain which manifest the decrease most, and whether a part of the decrease might result from migration of cells into or out of the wounded area. In fetal wounds, this question is less complicated, as there is no significant infiltration of inflammatory cells, and fibroblasts and epithelial cells/keratinocytes continue to comprise the main cellular populations. Our particular interest is in determining the mechanisms behind scarlessness versus scarring, and the ultimate end effector cells in this process must be fibroblastic [38], although of course subject to influence from other cell types. Accordingly, we based the current study on fibroblasts derived from rabbit fetal and adult skin and focused on the role of CCT-eta in fibroblast migration and cellular traction force, two key determinants of the fibrotic phase of dermal repair. Our own observations with immunohistochemical analysis of healing fetal and adult wound tissues indicate that fetal fibroblasts express little CCT-eta *in vivo*, whereas adult wound fibroblasts appear to stain more heavily, consistent with our *in vitro* results (Satish et al., manuscript in preparation).

Cell migration plays a vital role in wound healing, tissue morphogenesis, angiogenesis, and metastasis [39] and has been previously studied *in vitro* using a variety of methods, eg., on various mechanical substrates, 3-D vs. planar migration etc. Using a widely accepted planar migration assay we first explored whether the migratory capabilities of fetal and adult fibroblasts differed either at baseline or in response to growth factors implicated in wound healing. This question has yielded conflicting answers in previous studies: some suggest that fetal fibroblasts display enhanced migratory activity compared to the adult cells [40], [41], [42] whereas others do not [43]. It has been reported that serum can both stimulate [44] and inhibit [45] fetal and adult fibroblast motility; others find fibroblast migration is entirely independent of serum factors [39]. Complicating the picture, many investigators use fetal fibroblast cell lines rather than primary cell cultures [46], [47], [48].

Our results with low passage primary cultures of fetal and adult fibroblasts showed no difference in the basal motility of these cells, but demonstrated a markedly different response to the fibrogenic growth factors PDGF and EGF. Adult fibroblasts dramatically increased their migration in response to these agents but fetal fibroblasts remained insensitive to this stimulus. This distinct physiology may prove relevant to the differing wound conditions these cells might be expected to encounter: adult cells would presumably find it advantageous to migrate rapidly to seal off an exposed wound site, whereas fetal cells, programmed for function in the protected uterine environment, may not biologically recognize any such imperative.

To selectively manipulate CCT subunit levels in our cells of interest we used siRNAs, which proved effective in reducing the targeted mRNAs and proteins. The results presented here derive from the use of single siRNA constructs; we did undertake similar experiments with multiple siRNAs to attempt to maximize the effects seen, but the results were essentially identical to those with single constructs alone.

The reduction of CCT-eta with siRNA significantly reduced the basal and growth factor-induced migration of adult fibroblastic cells. This observation is in itself striking: although many hundreds of proteins are certainly involved in the complicated process of cellular locomotion, reduction of this one protein appears to have a significant biological effect. No such effect was seen when the CCT-beta subunit was decreased, suggesting that this may be a property specific to the eta subunit and distinct from its role as a component of the CCT holoenzyme. By reducing the adult fibroblasts' ability to respond to growth factor induction, it may be considered that selective downregulation of CCT-eta shifts the cells' functional phenotype toward a condition approximating that of fetal fibroblasts. The results presented here are for EGF and PDGF, but a similar pattern obtains also for transforming growth factor-beta, a highly pro-fibrotic agent which can be similarly counteracted in its effects on fibroblast locomotion by our siRNA constructs (Satish et al., manuscript in preparation). Thus CCT-eta may prove an attractive target through which the biological end effects of multiple scar-promoting growth factors and cytokines may be blocked concomitantly.

Another cellular characteristic highly pertinent to wound healing and scar formation is contractility, which we have assayed through measurement of applied cellular traction force (CTF); this assay has been found to correlate well with other established methods such as contraction of fibroblast populated collagen lattices [49]. We note that fetal fibroblasts exert less traction than adult, consistent with the disparate characteristics of healing fetal versus adult wounds. We note also that adult fibroblasts become even more contractile upon treatment with the growth factors EGF and PDGF, even as they similarly become relatively hypermotile. Although downregulation of CCT-eta does not appear to have any effect on cellular basal traction, it does appear to eliminate the ability of the cells to respond to a growth factor stimulus. The end result, as with cellular migration, is to shift the fibroblasts' behavioral profile in a direction more resembling that of fetal cells. Once again, CCT-beta has no such effect, highlighting again the distinctive importance of the eta subunit to this characteristic.

Although the above described observations demonstrate a clear role for CCT-eta in fibroblast physiology, and offer a rationale for why it is differentially regulated in fetal wounds and cells versus adult, they do not detail the exact molecular mechanism by which it acts. This question is a complicated one, since it is difficult to discriminate between the eta subunit's known role in the CCT holoenzyme and its potential functions independent of said holoenzyme. The CCT chaperonin complex itself stands at the nexus of a large network as it has been found to interact with a wide range of proteins implicated in such disparate cellular processes as endocytosis, mitochondrial function, and even chromatin remodeling, among others [50]; disruption of the CCT complex (such as might be accomplished by depletion of any given subunit) may therefore have pleiotropic effects. Of immediate apparent relevance is the role of CCT in the synthesis and maturation of actin, a protein essential to both motility and contractility. However, *in vitro* biochemical studies of the interaction between α-SMA and CCT have suggested that the beta subunit is more directly essential to this dynamic pairing than eta [51]. Per this previous evidence, then, we might expect to see a greater effect on α-SMA with CCT-beta siRNA than with CCT-eta; instead the opposite obtains. In fact, we observe effective inhibition of α-SMA using CCT-eta siRNA only, whereas cells treated with CCT-beta siRNA do not show a significant difference in α-SMA levels. This remarkable observation highlights the specific importance of the eta subunit to α-SMA expression (and the cellular characteristics which derive from it).

In fact, both cellular motility and contractility have previously been well connected to actin expression, and there is also evidence for a particular role for the α-SMA isoform. Tsai et al. [52] found that ultrasonic treatment of tendon-derived fibroblasts resulted in increased α-SMA and a corresponding increase in cell motility. Conversely, treatment of tendon fibroblasts with glucocorticoids diminished α-SMA expression with a correlated decrease in cell motility [53]. Our own findings are in accordance with these results, with an experimentally-induced specific reduction of α-SMA resulting in decreased fibroblast motility. Further, adult fibroblasts, with higher levels of both CCT-eta and α-SMA, were clearly better able to migrate in response to growth factor stimulation than the relatively eta- and α-SMA-deficient fetal fibroblasts.

There is a strong case as well for the importance of α-SMA to fibroblast contractility. Chen et al. [54] examined α-SMA expression and cellular traction force (CTF) in fibroblasts stimulated with TGF-β. They found that both α-SMA levels and CTF increased in a dose-dependent manner. The TGF-β-induced increase in CTF was reversed by an siRNA specific to α-SMA. Other agents that inhibited TGF-β-induced α-SMA expression (eg. basic fibroblast growth factor and a TGF-β type I receptor inhibitor) also resulted in reduced CTF. These observations were found to be unrelated to non-muscle myosin II and beta-actin expression, and clearly implicate α-SMA as an important mediator of fibroblast/myofibroblast contractility as well as motility.

In this context, and coupled with the previously noted finding that α-SMA expression correlates with contracting, scar-forming wounds but is absent from scarlessly healing wounds, our results build upon a growing body of evidence that fibroblast motility and contractility are important determinative features of wound contraction and scar formation, and that a critical element in this process is α-SMA. Since CCT-eta is shown herein to be essential for α-SMA protein accumulation, we posit that reduction of CCT-eta regulates fibroblast motility and contractility through its downstream reduction of α-SMA. The reduction of CCT-eta seen in fetal wound tissues, reflected also in the relative paucity of CCT-eta in fetal fibroblasts, may therefore be a crucial component of the scarless nature of fetal wound repair.

Although our findings demonstrate the specific importance of the CCT-eta subunit to fibroblast biology and define its effect on α-SMA, the question of whether this effect is through perturbation of CCT holoenzyme function or is a CCT-independent phenomenon remains unsettled. Since reduction of CCT-beta had no effect on fibroblast motility and contractility (but would be expected to disturb CCT holoenzyme function in a manner similar to CCT-eta depletion), it remains possible and perhaps even likely that our observations derive from a CCT-eta monomer-specific function.

CCT-eta has been shown to have a specific biologic activity separate from its role in the greater CCT complex: CCT-eta was recently found to be a co-factor for the soluble guanylyl cyclase (sGC), the chief intracellular secondary mediator of nitric oxide (NO) signaling [26]. Eta acts as an inhibitor of sGC; the net result of a downregulation of eta, therefore, would be a relative increase in sGC activity, resulting in increased cGMP and amounting to an increased functional NO signal. NO has previously been found to inhibit the PDGF-induced migration of fibroblasts and pericytes [55] and to inhibit the trans-endothelial migration of T cells [56], but likely acts in cell specific fashion through a variety of downstream molecular mediators. Although NO has been well studied in adult wound healing and is thought to favorably affect wound healing in general [57], [58], [59], its role in fibroblast physiology and especially in fetal wound repair is still relatively unexplored. The interaction of CCT-eta with sGC may represent one molecular mechanism which can (under certain circumstances) recapitulate an NO-dependent physiology.

Surprisingly, our results show that downregulating CCT-eta actually decreased cGMP levels in adult fibroblasts (data not shown). Since steady state cGMP levels are obviously the product of countervailing synthetic and degradative processes, it remains possible that decreasing CCT-eta is allowing for actual increased cGMP production but that this is overwhelmed by other downstream factors. On balance, however, with reduced cGMP levels it seems unlikely that the effects we observe by inhibiting CCT-eta on fibroblast contractility and motility are operating through a process that recapitulates nitric oxide signaling. Of course, both contractility and motility are extremely complex properties that result from the summation of numerous factors within cells; more experimentation will be required to further elucidate which other specific interactions may contribute particularly to fibroblast behavior and to the different properties of fetal versus adult cells.
